# Avian GIS models signal human risk for West Nile virus in Mississippi

**DOI:** 10.1186/1476-072X-5-36

**Published:** 2006-08-31

**Authors:** William H Cooke, Katarzyna Grala, Robert C Wallis

**Affiliations:** 1 Department of Geosciences/GeoResources Institute, Mississippi State University, MS, USA; 2 Michael Baker Jr., Inc. Natchez, MS, USA

## Abstract

**Background:**

West Nile virus (WNV) poses a significant health risk for residents of Mississippi. Physicians and state health officials are interested in new and efficient methods for monitoring disease spread and predicting future outbreaks. Geographic Information Systems (GIS) models have the potential to support these efforts. Environmental conditions favorable for mosquito habitat were modeled using GIS to derive WNV risk maps for Mississippi. Variables important to WNV dissemination were selected and classified as static and dynamic. The static variables included road density, stream density, slope, and vegetation. The dynamic variable represented seasonal water budget and was calculated using precipitation and evaporation estimates. Significance tests provided deterministic evidence of variable importance to the models.

**Results:**

Several models were developed to estimate WNV risk including a landscape-base model and seasonal climatic sub-models. P-values from t-tests guided variable importance ranking. Variables were ranked and weights assigned as follows: road density (0.4), stream density (0.3), slope (0.2) and vegetation (0.1). This landscape-base model was modified by climatic conditions to assess the importance of climate to WNV risk. Human case data at the zip code level were used to validate modeling results. All models were summarized by zip codes for interpretation and model validation. For all models, estimated risk was higher for zip codes with at least one human case than for zip codes where no human cases were recorded. Overall median measure of risk by zip code indicated that 67% of human cases occurred in the high-risk category.

**Conclusion:**

Modeling results indicated that dead bird occurrences are correlated with human WNV risk and can facilitate the assessment of environmental variables that contribute to that risk. Each variable's importance in GIS-based risk predictions was assigned deterministically. Our models indicated non-uniform distribution of risk across the state and showed elevated risk in urban and as well as rural areas. Model limitations include resolution of human data, zip code aggregation issues, and quality/availability of vegetation and stream density layers. Our approach verified that WNV risk can be modeled at the state level and can be modified for risk predictions of other vector-borne diseases in varied ecological regions.

## Background

West Nile virus (WNV) was detected in Mississippi in 2001 and still poses a significant risk for the state residents. Duration and frequency trends of WNV outbreak observed in Mississippi have corresponded closely to those of the continental United States. In 2002, the U.S. experienced the largest WNV epidemic ever recorded with 4,156 human cases and 284 deaths [1]. That same year, Mississippi also observed a record high number of human cases with 193 infections and 12 fatalities [2]. A year later in 2003, there were more than 9,000 human cases with 220 deaths nationwide [1]. In Mississippi, 83 humans tested positive and 2 deaths were recorded [3]. By 2004 the number of WNV infections in the U.S. decreased significantly. There were 2,470 human cases and 88 deaths, including 52 cases and 4 deaths in Mississippi [4,5]. The risk of WNV appears to have significantly lowered over time, but it is not clear whether this trend is due to changes in climate, increased public awareness or immunological responses. Most researchers agree that WNV is "not a passing phenomenon" and that it is difficult to predict the course of the disease over the coming years [6,7]. Modeling tools available in Geographic Information Systems (GIS) have the potential to accurately monitor the disease spread and enable prediction of future epidemiological trends.

The goal of this project was to estimate the likelihood of WNV infection in the state of Mississippi. This was achieved by analyzing avian and environmental data to model habitat suitability for mosquitoes that carry WNV. In Mississippi, there are numerous species of mosquitoes; however, only a few are believed to be responsible for WNV transmission to humans (Table 1). Among the most important are *Aedes aegypti*, *A. albopictus*, *Culex pipiens, C. quinquefasciatus*, *Ochlerotatus sollicitans*, *O. triseriatus*, and *Psorophora columbiae *[8,9]. These species inhabit a variety of environments and can be found in urban as well as rural settings. We viewed mosquito habitat suitability as a surrogate for estimating potential risk of WNV infection for humans and tested the usefulness of selected environmental variables in an analytical risk model.

**Table 1:** Important WNV mosquitoes of Mississippi [8,9].

Mosquito species	Habitat preference	Flight range	Host preference	Activity time	Life cycle to transmission
Yellow Fever *Aedes aegypti*	Shaded artificial containers, tree holes	200 m	Mammals	Crepuscular/day	10 – 20 days
Asian Tiger *Aedes albopictus*	Artificial containers, tire piles	200 m	Opportunistic	Crepuscular/day	10 – 20 days
Salt Marsh *Ochlerotatus sollicitans*	Salt marshes, freshwater	2500 m	Large mammals	Crepuscular/day	7 – 10 days
Tree Hole *Ochlerotatus triseriatus*	Artificial containers, tree holes	200 m	Mammals	Crepuscular/day	28 days
Southern House *Culex quinquefasciatus*	Waters heavily polluted with organic material	2000 m	Birds	Crepuscular/night	10 – 14 days
Common House *Culex pipiens*	Open, polluted high in organics water	2000 m	Birds	Crepuscular/night	10 – 14 days
Dark Ricefield *Psorophora columbiae*	Open freshwater temporary pools and ditches	At least 10 miles	Opportunistic	Day/Night	4–10 days

There were three important aspects of the study methodology. First, we assessed the WNV risk at a statewide scale. Statewide risk assessments offer the potential for optimization of mosquito spraying, allocation of educational materials, and sampling efforts. Second, we used environmental variables to identify areas ecologically capable of sustaining the virus. Finally, we developed an innovative way to construct risk predictions using raster-based GIS modeling.

A few published studies consider environmental aspects of WNV infection [10-12]. Most researchers rely on dead bird reports or mosquito data as an indicator that human cases will occur in the surrounding area [13-17]. Since avian (corvid) habitat distribution blankets the state, we believe that a bird-based model is too general to describe the coincidence of bird exposure to mosquito habitat and therefore we concentrate on the suitability of mosquito habitat. Another problem that impacts the usefulness of a bird-based model is the current sampling scheme. Bird testing varies by county, and generally when bird cases of WNV are diagnosed in the laboratory, there is little or no successive testing of dead birds. Consequently, the dead bird data only signals presence or absence of WNV, not the severity of the outbreak. For these reasons, bird data alone may not adequately characterize the pattern of WNV infection. However, bird data did play a vital role in our models since the spatial depictions of WNV bird infections provided the baseline data necessary to correlate environmental variables with virus infections.

Our approach is similar to studies that use environmental variables within GIS to model risk of other vector-borne diseases such as Lyme disease and Malaria [18-20]. Often, these models assign variable significance heuristically, based on expert opinions. For this study we determined variable significance and weights through a deterministic algorithmic approach with variable ranking assigned using statistical probability levels.

## Methods

### WNV data and preliminary spatial analyses

The study area comprised the entire state of Mississippi. Data records were summarized and analyzed within unique zip code boundaries. Data on laboratory-diagnosed human and bird cases recorded in years 2002 and 2003 were obtained from the Mississippi Department of Health. Data records included date of occurrence, zip code, and city name.

Each zip codes' centroid was calculated to enable spatial depictions of human and bird occurrences necessary for GIS-based spatial analyses. Spatial patterns of case occurrences were tested using Pearson's sample correlation coefficient (*r*) and quadrat analysis. Correlation analyses provided information about relationships between human population and bird and human case occurrences, while quadrat analysis provided information about tendency of cases to occur as spatially random, uniform or clumped.

**Figure 1:**
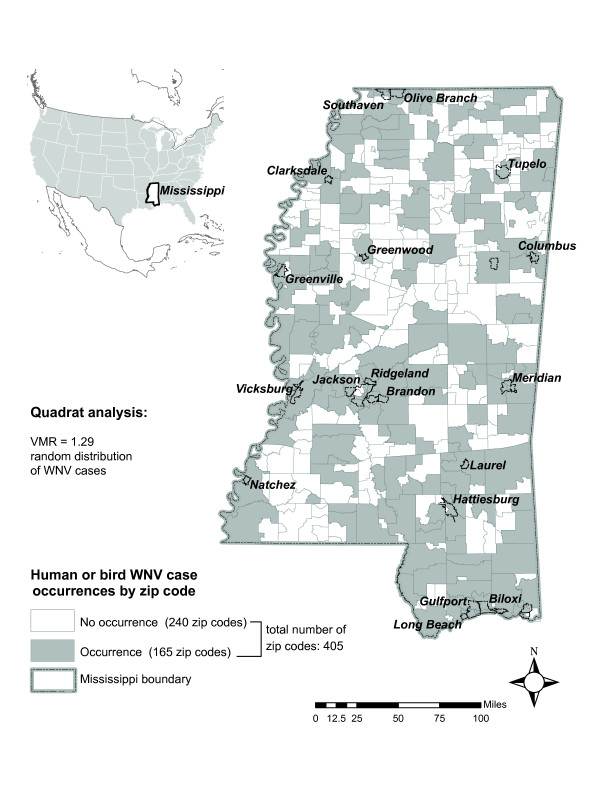
**Study area context map**. Areas of WNV infections (combined human and bird data) in Mississippi in 2002 and 2003.

A considerable number of zip codes (165 out of 405) reported at least one human or bird WNV infection in years 2002 and 2003 (Figure 1). We examined the pattern of combined human and bird infections in the state. Quadrat analysis indicated a tendency towards a random distribution of cases [variance to mean ratio (VMR) = 1.29]. In general, when VMR approaches zero the distribution is uniform, a VMR near 1 indicates a random distribution, while VMR near 2.0 indicates a tendency toward clustered distribution [21].

**Figure 2:**
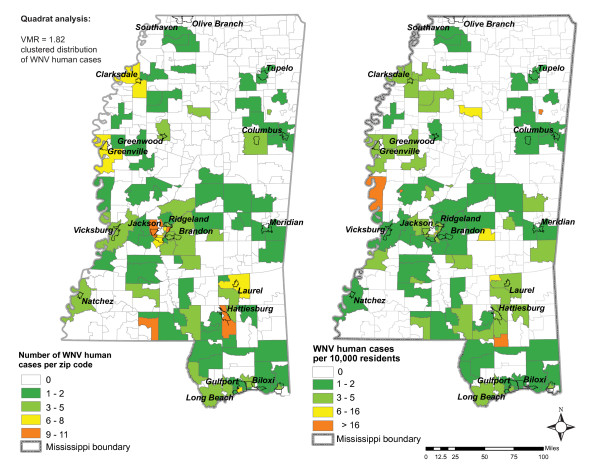
**WNV human cases in 2002 and 2003**. Occurrences categorized by number of cases per zip code (map on left) and WNV human cases normalized by population (number of WNV cases per 10,000 residents) (map on right).

Positive human cases of WNV in 2002 and 2003 indicated tendency towards a clustered distribution (VMR = 1.82) with cases diagnosed in 104 out of 405 zip codes (Figure 2). Large clusters of zip codes with diagnosed WNV human cases are located in southern and central part of Mississippi, whereas smaller and more isolated clusters are scattered over the northern part of the state. Only one or two cases were diagnosed in most (64%) zip codes where human cases of WNV occurred. More serious outbreaks (more than 3 cases) were observed in 29 zip codes. Most zip codes with a high number of positive human cases are densely populated. These areas are located close to large metropolitan centers such as Jackson, Hattiesburg, Clarksdale, and Greenville, which seems to indicate an urban population bias. However, normalizing case count by population revealed that the main outbreak centers are actually located in rural areas (Figure 2). Non-significant correlation (*r *= 0.094) between number of human cases and human population provided further indication that a population bias does not exist.

**Figure 3:**
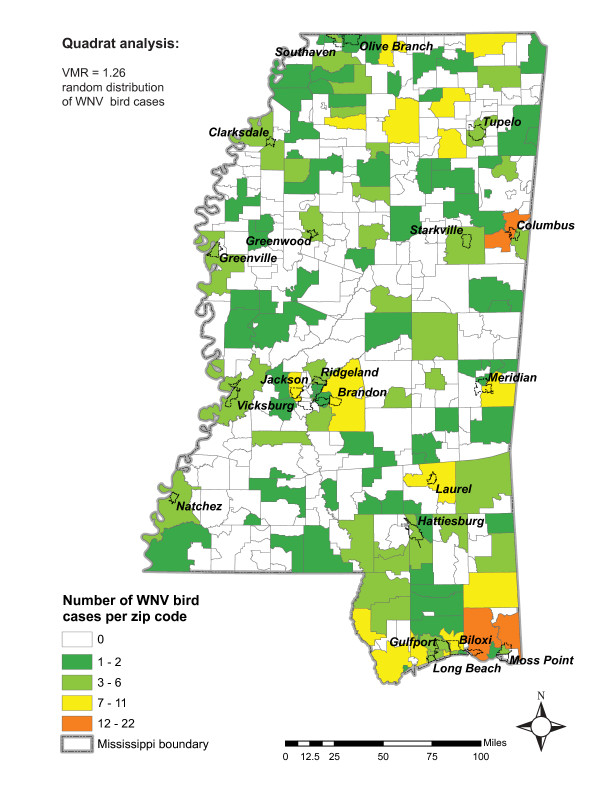
WNV bird cases in 2002 and 2003 categorized by number of cases per zip code.

Patterns of positive bird cases are more difficult to interpret spatially. At least one confirmed WNV bird case occurred in 137 zip codes. A quadrat analysis indicated that cases within these zip codes tended towards a random distribution (VMR = 1.26) (Figure 3). While many studies use dead bird data to model WNV, these data are potentially biased [14,15,22]. A population density bias was expected because a person must find a dead bird and bring it in for testing. As Figure 3 shows, a larger number of bird infections appears to cluster in zip codes that have high population density. However, no significant correlation between number of bird cases and human population (*r *= 0.015) was observed. Consequently, geographic locations of high population density do not appear to bias the discovery of dead birds.

**Figure 4:**
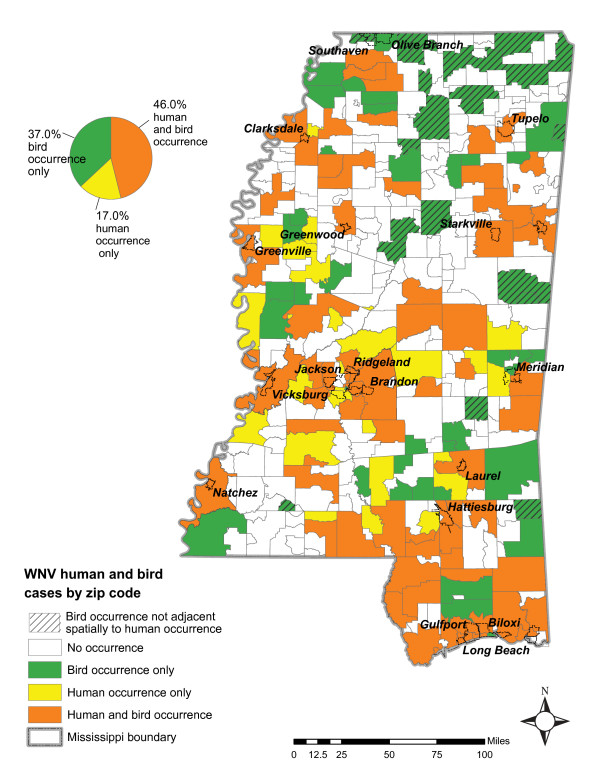
**Areas of WNV infections in 2002 and 2003**. Infections categorized by the type of occurrence: bird occurrence only, human occurrence only, human and bird occurrence.

Interestingly, there appears to be no correlation between the number of human infections and the number of bird infections per zip code. However, the presence of bird case occurrences and human case occurrences per zip code were positively correlated (*r *= 0.488), indicating that there is a strong spatial relationship between the coexistence of human and bird WNV occurrences (Figure 4). Both human and bird cases occurred in 46% of infected areas. Also, the majority of zip codes that had human cases but no bird cases were adjacent to areas of bird infections.

These preliminary analyses indicated that the dead bird occurrence data signals the presence of WNV and provides a fairly random sample of WNV infections on the Mississippi landscape. This enabled tests for the environmental variable states that may be related to human infections.

### Variables

Review of the literature on vector-borne disease modeling led to the conclusion that numerous environmental factors might be critical to WNV dissemination. The modeling approach and variables used in our research are similar to other GIS-based studies that assess environmental risk factors for Lyme and Malaria diseases using information on land use, land cover, forest distribution, soils and elevation [18-20].

Other researchers have also used these and similar variables in estimation approaches to assess WNV risk [10-12]. There are numerous recent examples of WNV risk modeling employing a variety of environmental variables. For example, Ruiz et al. (2004) used several factors related to the physical environment such as elevation range, physiographic region, and percentage of vegetation cover to determine WNV risk during 2002 outbreak in the Chicago area. Tachiiri et al. (2006) developed a raster-based model using basic geographic and temperature data to assess WNV risk in British Columbia. Gibbs et al. (2006) determined that temperature, housing density, urban/suburban land use, and physiographic region are important variables affecting the geographic distributions of WNV in the state of Georgia.

The selection of environmental variables used in our study was based on evaluation of specific Mississippi mosquito habitat conditions and statewide raster data availability. We determined that slope, road density, stream density, vegetation, aspect, soil permeability, and climatic variables should be tested for their importance in modeling infection risk. Although some multi-colinearity may exist among these variables, the goal of this research was to produce a spatial estimation of WNV risk to humans. Since estimation was the goal, validated map accuracy of estimated human risk superseded concerns of variable multi-colinearity.

Variables were maintained as raster grids with 120 m cell resolution and divided into two states: static and dynamic. Static variables are assumed to change slowly or not at all over time. Slope, road density, stream density, and vegetation were selected to model static landscape conditions on the basis of significance tests. The slope percent layer was generated from a statewide 30 m Digital Elevation Model (DEM). The 2002 TIGER files from the Census Bureau were used to create the road density grid. The stream density layer was derived from merged data including major rivers, perennial streams, and intermittent streams. These data were acquired from the Mississippi Automated Resource Information System (MARIS). A GIS 'Kernel' density function was used to generate both road and stream density layers. This function approximates a Gaussian distribution that assigns greater importance to values near the 'kernel' center and was implemented in ArcGIS Spatial Analyst. The radius chosen for each density calculation was selected using an iterative process that maintained local variation without over-generalizing density estimates for each variable. The resulting continuous-field surfaces show the density of roads and stream networks throughout the state. The 120 m resolution surfaces provided site-specific information for each grid cell that was further summarized by zip code to assess the WNV risk. Vegetation was derived from Moderate Resolution Imaging Spectroradiometer (MODIS) 16-day Normalized Difference Vegetation Index (NDVI) composites at 250 m resolution. The NDVI is a normalized ratio of red and near infrared wavelengths commonly used to estimate vegetative cover [23]. The data representing April 7–23, 2002 were downloaded from the Land Processes Distributed Active Archive Center (LPDAA) [24]. MODIS data were resampled to 120 m grid using a centroid to point conversion followed by an Inverse Distance Weighting (IDW) interpolation. Some data smoothing occurred during this process. Although NDVI changes over time, we treated it as a static variable. Using NDVI as a dynamic variable is problematic for several reasons. The native 250 m resolution requires resampling to the higher resolution of other variables, which may introduce uncertainty into the model. The NDVI compositing process can result in large off-nadir view angles particularly in light of the high incidence of cloud and haze during the summer months. Also, the compositing process often fails to yield cloud-free imagery over desired temporal periods associated with mosquito life cycles. This was a major problem for 2002, which was the second wettest summer/fall period in 108 years of climate records. Cloud-free near-nadir NDVI mosaics that were available for most of the summer and fall were generally of poor quality. Finally, vegetation response to rainfall events has variable site/species lag times that are more accurately characterized by the climatic variable.

Careful consideration was given to climate as the dynamic variable in our study. In collaboration with Dr. C. Wax, (MS State Climatologist), the influence of water budget on mosquito populations was considered. The water budget [Precipitation minus Evaporation (P-E)] is a meaningful and critical measure of water that is available in the environment to support mosquito breeding. The literature suggests that *Culex *species breed preferentially in organically rich water associated with decreased breeding areas while *O. sollicitans *mosquitoes prefer fresh water conditions associated with increased breeding areas [9]. What is not clear in the literature is whether increased or decreased breeding area influences risk of WNV infections. It is possible that decreased breeding areas consistent with low P-E (drought) places urban dwellers at increased risk while increased breeding areas consistent with high P-E values (water surplus) places rural dwellers at increased risk. As a consequence of this water-dependent breeding behavior, both luxury rainfall and drought conditions can result in risk conditions that affect different geographical areas with diverse population patterns. Our research tried to answer the question, how does climate impact WNV? We assessed the importance of climate to WNV human occurrence by varying the weighting factor of climate in the GIS WNV risk prediction models.

Precipitation (P) and evaporation (E) data for 2002 and 2003 were used to characterize climate. Precipitation minus evaporation (P-E) was a derived variable that represented water balance in the environment. Precipitation (P) was derived from Multi-Sensor Precipitation Estimates (MPE) by interpolating ground-bias corrected Doppler Weather radar point data. Interpolation between the 4 km Hydrologic Rainfall Analysis Project (HRAP) grid points was performed using the IDW approach. IDW provides reasonable estimates of precipitation for summer and fall and is much easier to implement operationally than methods like 'Kriging' that require interpreter analysis of semi-variograms for each interpolation period [25]. Evaporation is considered to be a fairly constant element with most values occurring in an interval between 2.54 mm and 7.62 mm durring the summer-fall period. Evaporation is also more spatially homogeneous than precipitation [26]. The spatial homogeneity of evaporation lends itself well to a splining interpolation technique that uses mathematical functions that describe smoothly curving features and can result in a close fit with relatively few control points (typical of pan evaporation data) [27]. The interpolated data resulted in a continuous-field layer of evaporation that matched the resolution of the existing continuous-field layer of precipitation. Subtracting daily evaporation from precipitation produced a dynamic GIS layer that expressed a moisture condition suitable for GIS models.

**Figure 5:**
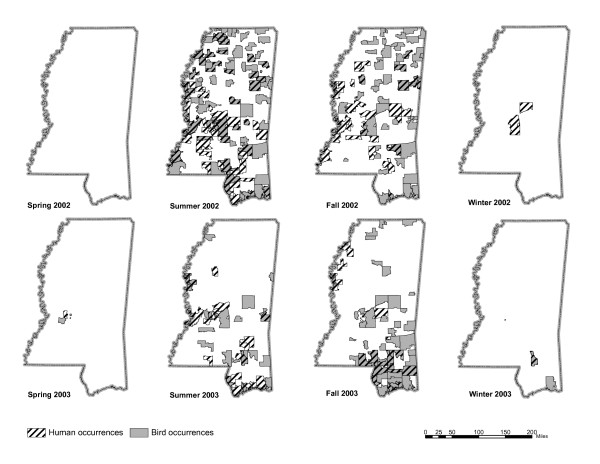
Seasonal bird and human WNV occurrence data.

Due to lack of sufficient human case validation data at shorter temporal intervals, seasonal summaries were chosen to provide adequate sample size for calculating the climatic variable influence. Since the majority of WNV cases occurred between June and November (Figure 5), the models were constructed for summer and fall only.

### Methodology

**Figure 6:**
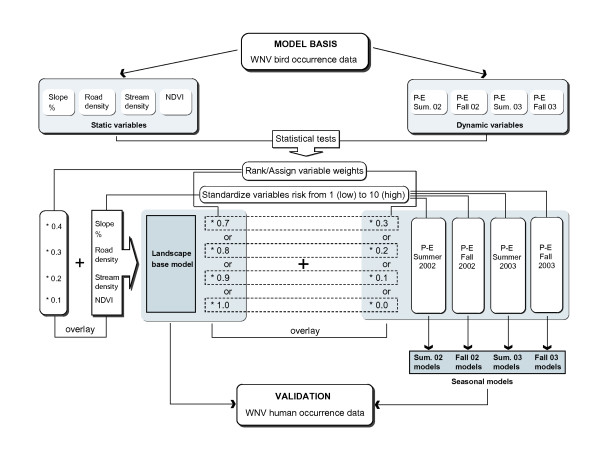
Methodology flowchart.

The spatial distribution of bird infections provided test locations critical for determining whether correlations exist between environmental variables and disease incidence. We used a simple additive weighting (SAW) method for model construction and made several modeling assumptions [28]. We assumed that: people become infected within the zip code of their residence, selected environmental factors are related to the WNV outbreak, and probability of human infection is higher in zip codes with confirmed WNV bird cases. Variable correlations between bird infections and environmental conditions, variable importance values, and the model development process are detailed in Figure 6. Note that human occurrence data was used to validate models and to test for climatic influences on the spatial distribution of the disease. Human data for the years 2002 and 2003 were used to test model prediction accuracies.

Environmental variables were all converted to raster format and subset to the boundary of Mississippi. All data were transformed to a common map projection, and resampled to 120 m grid cell resolution. Statistical tests were designed to establish how selected variables correlate to WNV bird case occurrences and which variables are the most significant on the basis of "difference in means test" (t-test). For each variable, mean responses for infected versus non-infected zip codes were compared at the 95% confidence level. Statistical tests were performed with non-weighted occurrences, representing either presence or absence of a positive bird case, rather than the number of dead birds reported by zip code, since the correlation analyses indicated a non-significant relationship between human population and number of WNV avian infections. Results of these tests helped to determine the importance rank of each environmental variable in the risk models. Ranks were established based on the t-test results: the more significant t-test result the higher rank assigned. Once the ranking was assigned, numerical weights were calculated according to the following formula [28]:

wj=n−rj+1∑ (n−rk+1)
 MathType@MTEF@5@5@+=feaafiart1ev1aaatCvAUfKttLearuWrP9MDH5MBPbIqV92AaeXatLxBI9gBamXvP5wqSXMqHnxAJn0BKvguHDwzZbqegyvzYrwyUfgarqqtubsr4rNCHbGeaGqiA8vkIkVAFgIELiFeLkFeLk=iY=Hhbbf9v8qqaqFr0xc9pk0xbba9q8WqFfeaY=biLkVcLq=JHqVepeea0=as0db9vqpepesP0xe9Fve9Fve9GapdbaqaaeGacaGaaiaabeqaamqadiabaaGcbaGaem4DaC3aaSbaaSqaaiabdQgaQbqabaGccqGH9aqpdaWcaaqaaiabd6gaUjabgkHiTiabdkhaYnaaBaaaleaacqWGQbGAaeqaaOGaey4kaSIaeGymaedabaacceGae8xeIuUaaGPaVlabcIcaOiabd6gaUjabgkHiTiabdkhaYnaaBaaaleaacqWGRbWAaeqaaOGaey4kaSIaeGymaeJaeiykaKcaaaaa@543A@

Where:

*w*_*j *_is normalized weight for the *j*^*th *^criterion

*n *is the number of criteria under consideration (k = 1,2,...*n*)

*r*_*j *_is the rank position of the criterion

Modeling using SAW method requires standardization of variable value ranges. We categorized each variable's values into 10 classes with 1 representing lowest risk and 10 representing highest risk. The "quantile" classification method was used. With the "quantile" method, the range of possible values is divided into certain number of intervals (classes), so that the each class contains the same number of features. This classification method is useful to emphasize changes in the middle values of the distribution, because the intervals are usually wider at the extremes [29].

It is important to note the landscape-base model was created using static variables only, and served as the baseline for seasonal models. Four seasonal sub-models were created varying the proportional contribution of climate in the models to assess the importance of climate in the estimation of the WNV risk.

## Results and discussion

### Landscape-base model

The landscape-base model was constructed with the assumption that mosquito habitat suitability factors can be used to estimate the risk of WNV. We disregard the potential multi-colinearity among these factors and assume that all are important to the WNV risk estimation. The results of the statistical tests used to deterministically assign ranks and compute weights for the landscape variables are shown in Table 2.

**Table 2:** Summary of static variable testing, standardization, model ranking and weight calculations.

Variable	Relation to ecology of WNV vector mosquitoes	Mean for zip codes with		T-test significance	WNV risk level	Variable	
					
		WNV bird occurrence	no WNV bird occurrence	(p-value)	1 – low risk 10 – high risk	Rank	Weight
Road density	Breeding sites along roads	1.7568	1.1550	.001	High rd. 10 Low rd. 1	1	0.4
Stream density	Water as habitat	1.1200	1.1868	.010	High sd. 1 Low sd. 10	2	0.3
Slope percent	Aspect of water outflow rate	7.1416	7.9886	.028	Gentle sl. 10 Steep sl. 1	3	0.2
NDVI vegetation	Vegetation as resting and breeding sites	164.6797	160.9131	.251	High NDVI 10 Low NDVI 1	4	0.1

The significance test p-value for each variable was the basis for ranking variable importance. Variables were ranked and weights assigned from most important to least important as follows: road density (0.4), stream density (0.3), slope (0.2) and NDVI (0.1).

The test for equality of means for road density resulted in a significant p-value (p < 0.001) indicating that road density for zip codes of WNV occurrence is significantly different from zip codes of non-occurrence. A review of the means indicates that higher values of road density are related to the WNV occurrence and increased risk. These results of statistical testing are supported by mosquito habitat studies. For example, *C. pipiens*, often considered the principal carrier of WNV, prefers to breed in human-created environments. Features of a road system such as culverts, storm drains and roadside ditches easily become ideal mosquito-breeding sites, especially when clogged and polluted [9].

A significant p-value (0.010) for stream density indicates that stream density is different for zip codes of WNV occurrence versus zip codes of non-occurrence. Comparison of the means indicates that the lower values of stream density are related to WNV occurrence and higher risk. Stream density is generally low in large flood plains (Mississippi River), but is relatively high in other river floodplains (Pascagoula, Tenn-Tom, etc). This is due to the confluence of minor streams, multiple channels (braided characteristics of southern Mississippi streams flowing through sandy substrate), and meandering stream characteristics. Running water and the flushing effect of large rain events on the floodplains favors fresh water breeding mosquito species (O. *sollicitans, P. columbiae*).

A significant p-value (0.028) for slope percent indicates that mean slope is statistically different for zip codes of WNV occurrence versus zip codes of non-occurrence. Comparison of the means indicates that gentle slopes are related to increased WNV risk. Water is more likely to pond on gentle slopes, resulting in favorable mosquito habitats.

Comparison of the means for NDVI (vegetation) indicates that zip codes with WNV occurrence have slightly higher NDVI values than zip codes with no WNV occurrence. The p-value (0.251) suggests that there is essentially no statistical difference between NDVI levels for zip codes with WNV occurrence and those with the absence of WNV cases. Literature suggests that vegetation is associated with mosquito habitat. Many mosquito species use vegetation as resting sites, while some species such as *O. triseriatus *utilize tree holes for breeding purposes [30]. It is therefore surprising that NDVI was not statistically correlated to WNV infections. We suspected that this might be due to the fact that the spring NDVI image did not coincide with vegetation phenology important for resting behavior of mosquitoes.

To summarize, in Mississippi WNV risk appears to be associated with high road density, low stream density, and gentle slopes. NDVI was included in the model since it is suggested in the literature that vegetation is highly correlated with mosquito breeding habitat. The linear additive model for risk prediction was constructed as follows:

[road density] * 0.4 + [stream density] * 0.3 + [slope %] * 0.2 + [NDVI] * 0.1

**Figure 7:**
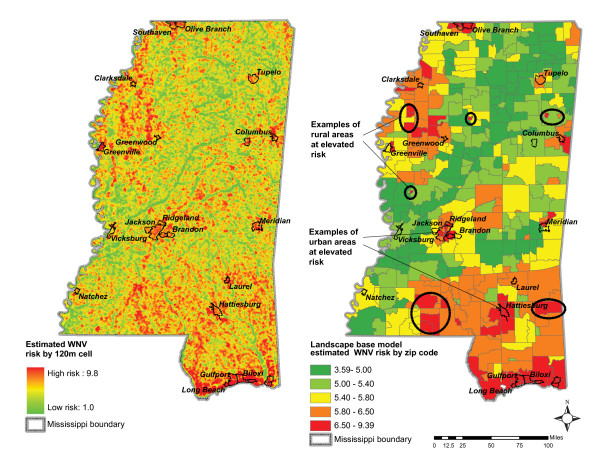
**Landscape-base model**. Results by 120 m cell (map on left); estimated WNV risk median values summarized by zip code (map on right).

Results of the landscape-base model are presented in Figure 7. Clearly, WNV risk is not uniform across the entire state. Validation of the risk models using human cases revealed that urban as well as rural areas might be at elevated risk.

### Seasonal models

Numerous studies discuss the relationship between climate and WNV infections [31-35]. Some authors advocate that such a relationship exists and that climatic data can be used to predict geographic spread of the disease as well as to specify the location of the outbreaks [33,36]. Consequently, one of the objectives of our study was to examine the climatic aspects of WNV outbreaks in Mississippi.

A recent hypothesis links the emergence of WNV in human populations with mild winters, prolonged periods of hot, dry spring weather followed by a significant rain events [32]. This hypothesis is also known as 'drought hypothesis' and many facts appear to reinforce it [32]. However, this hypothesis explains only general climatic trends associated with the WNV outbreaks and also appears to contradict the commonly accepted assumption that outbreaks of mosquito-borne diseases are amplified by wet weather conditions. Further, it can be argued that due to the major differences in mosquito habitat preferences both extremes could contribute to the outbreaks (Table 1). For example, *O. sollicitans *mosquito breeding conditions require rain and warm weather, while *A. aegypti *or *A. albopictus *mosquitoes can survive long dry periods and low temperatures [9].

Clearly, there are many interconnected ecological factors that need to be considered and there are many uncertainties about how climate contributes to the WNV outbreaks. For these reasons the use of climate data was particularly challenging in this project. We used an average seasonal balance between precipitation and evaporation in the analyses. In our opinion, this combined variable (P-E) gives a good representation of the environmental water balance. Seasonal risk models were generated on a basis of the landscape-base model overlaid with climate data. These models estimated the risk for summer 2002, fall 2002, summer 2003, and fall 2003.

**Figure 8:**
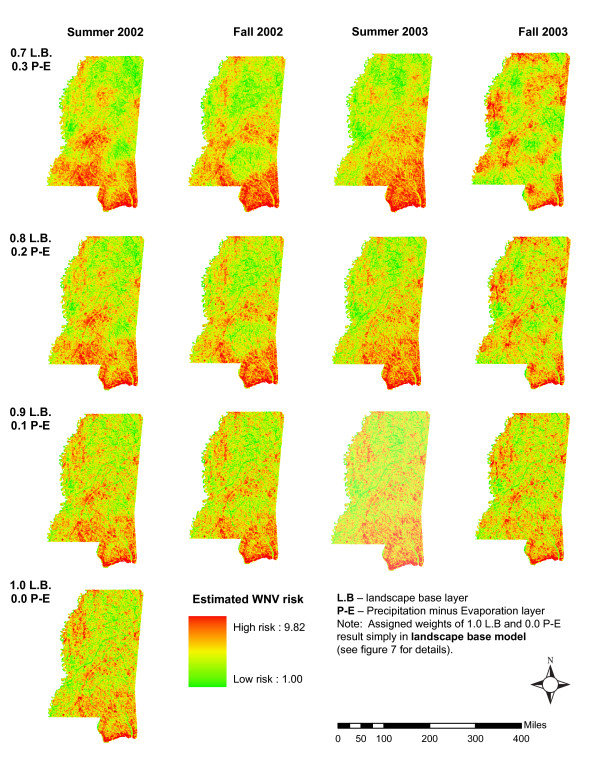
Results of seasonal models.

T-tests for climatic variables resulted in insignificant p-values for three out of four P-E seasonal variables. Only the results for summer 2003 P-E variable indicated significant difference between zip code classes. A review of the means for summer 2003 (1.1303 – occurrences and -0.8574 – non-occurrence) indicated that higher values of P-E are associated with increased risk of WNV occurrence. Results for summer 2003 appear to support the assumption that moist conditions contribute to the WNV outbreaks. The results for other seasons are inconclusive. Therefore, four seasonal models were created to investigate whether the addition of climate data could improve the estimation of the risk level. To test this objective each seasonal model was divided into four sub-models. The sub-models were created by varying the proportional contribution of climate to the landscape-base model (Figure 8). Using this method we were able to examine each model's 'sensitivity' and determine the significance of inclusion of climate data in the seasonal models. Results of the seasonal models are presented in Figure 8. Varying the proportional contribution of climate in the models resulted in substantial changes in the final risk maps.

### Models comparison and validation

The final WNV risk models were summarized by zip codes for easier interpretation of results and model validation. The landscape-base model was validated with combined human infection data for years 2002 and 2003. Dynamic models were validated with human infection data for 2002 and 2003 in the appropriate season.

Table 4 summarizes and compares the models, presents the risk estimates and validation results. The risk estimates were categorized by zip codes of human WNV occurrence and non-occurrence. Majority, mean, and median measures were compared for their usefulness in estimating risk. The average estimated risk was compared for zip codes with human cases versus zip codes with no human cases and differences recorded. The results indicated that for all models, estimated risk is significantly higher for zip codes with at least one human case than for zip codes where no human cases were recorded. These results indicate that bird occurrence data are correlated with human WNV risk and can facilitate the assessment of environmental conditions that contribute to that risk. Examination of differences in measures of central tendency indicates that the median (66.9 %) and majority (66.8%) reflects the percentage of WNV human cases in the high-risk category better than the mean (61.3%) (Table 4).

**Table 3:** Summary of t-tests for dynamic precipitation minus evaporation in inches (P-E) variables.

P-E variable by season	Mean for zip codes with		T-test significance (P-value)
		
	WNV bird occurrence	No WNV bird occurrence	
Summer 2002	-1.1492	-1.2772	.515
Fall 2002	4.3486	4.1149	.224
Summer 2003	1.1303	-0.8574	.001
Fall 2003	-1.182	0.0585	.080

**Table 4:** Summary table; results comparison and validation.

Model	Variables/weights	Validation WNV data	Majority Risk				Mean Risk				Median Risk			
			
			0	1	# hc	val.	0	1	# hc	val.	0	1	# hc	val.
			
			**difference**		**% correct**		**difference**		**% correct**		**difference**		**% correct**	
Landscape -base	Road den. – 0.4 Stream den. – 0.3 Slope % – 0.2 NDVI – 0.1	Human cases in 2002 and 2003	5.4 **1.0**	6.4	65 **62.5**	104	5.5 **0.7**	6.2	65 **62.5**	104	5.5 **0.7**	6.2	67 **64.4**	104

Summer 2002	Land. base – 0.7 P-E – 0.3	Human cases in Sum. 2002	5.3 **1.0**	6.3	47 **61.8**	76	5.4 **0.8**	6.2	47 61.8	76	5.4 0.9	6.3	46 60.5	76
	Land. base – 0.8 P-E – 0.2		5.4 **0.9**	6.3	45 **59.2**	76	5.5 0.7	6.2	50 65.8	76	5.5 0.7	6.2	49 64.5	76
	Land. base – 0.9 P-E – 0.1		5.4 **0.8**	6.2	46 **60.5**	76	5.5 **0.6**	6.1	48 **63.2**	76	5.5 **0.7**	6.2	50 **65.8**	76
	Land. base – 1.0 P-E – 0.0		5.5 **0.8**	6.3	44 **57.9**	76	5.6 **0.5**	6.1	44 **57.9**	76	5.5 **0.3**	5.8	46 **60.5**	76

Fall 2002	Land. base – 0.7 P-E – 0.3	Human cases in Fall 2002	5.5 **0.4**	5.9	20 **60.6**	33	5.5 **0.3**	5.8	16 **48.5**	33	5.5 **0.3**	5.8	16 **48.5**	33
	Land. base – 0.8 P-E – 0.2		5.5 0.5	6.0	20 **60.6**	33	5.6 0.2	5.8	16 48.5	33	5.6 0.2	5.8	16 48.5	33
	Land. base – 0.9 P-E – 0.1		5.6 **0.5**	6.1	18 **54.5**	33	5.6 **0.3**	5.9	15 **45.5**	33	5.6 **0.3**	5.9	15 **45.5**	33
	Land. base – 1.0 P-E – 0.0		5.6 **0.5**	6.1	19 **57.6**	33	5.6 **0.3**	5.9	17 **51.5**	33	5.7 **0.3**	6.0	18 **54.5**	33

Summer 2003	Land. base – 0.7 P-E – 0.3	Human cases in Sum. 2003	5.4 **1.4**	6.8	20 **66.7**	30	5.4 **1.3**	6.7	21 **70.0**	30	5.5 **1.2**	6.7	21 **70.0**	30
	Land. base – 0.8 P-E – 0.2		5.4 **1.4**	6.8	23 **76.7**	30	5.5 **1.2**	6.7	25 **83.3**	30	5.5 **1.2**	6.7	24 **80.0**	30
	Land. base – 0.9 P-E – 0.1		5.5 **1.4**	6.8	22 **73.3**	30	5.5 **1.2**	6.7	25 **83.3**	30	5.6 **1.2**	6.8	25 **83.3**	30
	Land. base – 1.0 P-E – 0.0		5.6 **1.3**	6.9	24 **80.0**	30	5.6 1.1	6.7	24 **80.0**	30	5.6 **1.2**	6.8	25 **83.3**	30

Fall 2003	Land. base – 0.7 P-E – 0.3	Human cases in Fall 2003	5.6 **1.0**	6.6	16 **59.3**	27	5.7 **0.7**	6.4	16 **59.3**	27	5.7 **0.8**	6.5	16 **59.3**	27
	Land. base – 0.8 P-E – 0.2		5.6 **1.1**	6.7	18 **66.7**	27	5.6 **1.0**	6.6	18 **66.7**	27	5.6 **1.0**	6.6	18 **66.7**	27
	Land. base – 0.9 P-E – 0.1		5.5 **1.4**	6.9	24 **88.9**	27	5.6 **1.1**	6.7	23 **85.2**	27	5.6 **1.2**	6.8	23 **85.2**	27
	Land. base – 1.0 P-E – 0.0		5.6 **1.4**	7.0	24 **88.9**	27	5.6 **1.2**	6.8	25 **92.6**	27	5.6 **1.3**	6.9	26 **96.3**	27

	Average % correct for all models				**66.8**				**61.3**				**66.9**	

The risk estimates by zip code were calculated using a zonal function. Majority, mean and median measures of risk were calculated and recorded for zip codes of WNV human occurrence (1) and zip codes of non-occurrence (0). Difference between the two categories of zip codes (0 versus 1) was determined and for all models. Estimated risk was higher for zip codes with at least one human case than for zip codes where human cases were not recorded. Modeling results were validated with human infection data (val.) for appropriate corresponding season. Number of human cases (#hc) in the high-risk category was determined. The high-risk category included zip codes of the combined top two risk classes (out of five) defined using quantile classification method. For each model percentage correct (% correct) was calculated to determine the measure of central tendency that works best for the validation. Examination of average % correct for all models indicated that the median (66.9%) and majority (66.8%) measures reflect the actual WNV risk better than the mean (61.3%).

Addition of climatic data improved risk estimation for summer models but worsened risk estimation for fall models. Summer models were not improved when the landscape-base/climate ratio exceeded 0.8/0.2. The ratio reached an optimum at 0.8 for landscape-base layer and 0.2 for P-E layer.

In general, 2003 models estimated WNV risk better than 2002 models. This might be associated with the fact that 2002 outbreak was considerably more severe, widely spread, occurred during a period of excessive rainfall, and therefore risk was more difficult to assess. The National Climatic Data Center reported that the fall of 2002 was the 107th wettest year in 108 years that data have been recorded. We believe that such a surplus of water in the environment makes the importance of water budget difficult to assess in this unusual year. In 2003, with near average precipitation, WNV case numbers decreased and were more spatially clustered. We assume that these clusters of cases were located around areas environmentally predisposed to sustain the illness. Therefore, the results of 2003 models might provide better risk estimation. We believe that models developed for other locations and in Mississippi during 'normal' years will benefit from the inclusion of climatic data. This is also borne out by other researcher's conclusions [10]. It appears, however, that severe outbreaks of mosquito-borne diseases might be difficult to predict and that prospective modeling efforts should consider additional factors such as socio-economic and demographic indicators if address-specific case data are available.

**Figure 9:**
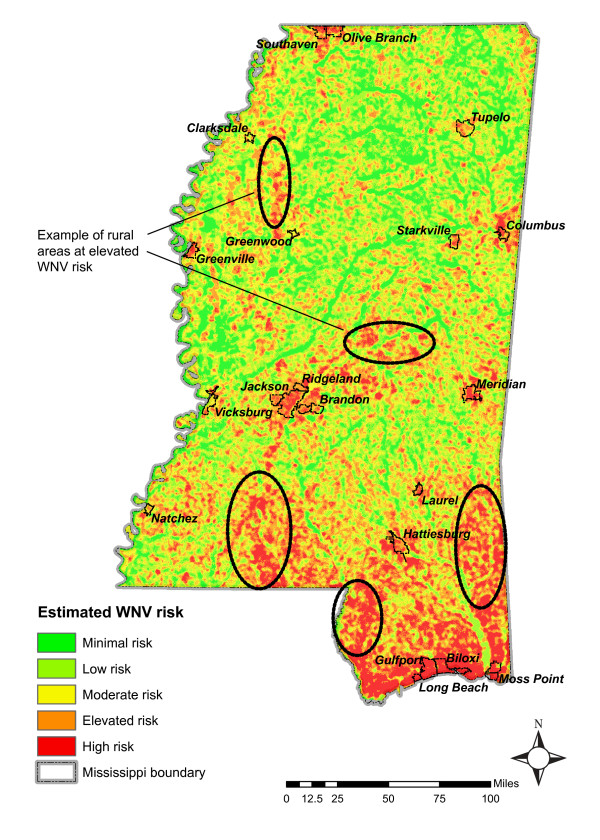
Summarization of risk across all seasons indicating areas environmentally prone to sustaining the WNV.

Summarizing risk for all seasons shows that in Mississippi environmental areas prone to sustaining the WNV include the Gulf Coast, Jackson metropolitan center, Hattiesburg, Meridian and Columbus, as well as numerous rural communities across the state as shown in Figure 9.

## Conclusion

In this study, spatial estimation of WNV risk in Mississippi was carried out by analyzing avian and environmental data to develop mosquito habitat suitability models. Several environmental factors were considered and according to our analysis, in Mississippi, WNV risk is correlated to high road density, low stream density, and gentle slopes. The methodology developed for this project is simple and can be easily modified for this and other vector-borne diseases in varied ecological regions. Bird-based WNV risk maps were validated with human case data and clearly show areas environmentally prone to sustaining the virus.

Heuristic methods of variable weighting often employed in GIS analyses can introduce personal bias in the modeling process. In our study, statistical tests of environmental variable significance provided deterministic evidence of each variables' importance (weight) for predicting risk using GIS. This approach diminishes the possibility of introduction of analyst' bias into the models.

The usefulness of selected static environmental variables (road density, stream density, slope, vegetation) to assess WNV risk was successfully demonstrated. The landscape-base model employing these four environmental variables was validated with human occurrence data and indicated geographic regions of increased infection risk to humans. The overall usefulness of the dynamic climatic variable into the models was uncertain. Both 2002 and 2003 summer models improved with the inclusion of climatic data, but the landscape-base model (without climate) was superior for both fall models. More research is needed to explore climatic data contributions to WNV risk prediction and to define the role of climate in the disease transmission process. For example, cumulative precipitation estimates like those utilized in fire prediction models (Keetch-Byram Drought Index) may be effectively incorporated. Currently, we are testing more temporally sensitive models that use daily calculations of cumulative P-E (as a departure from average) to represent water budget in the environment at any point in time. Ongoing fire risk studies have used this approach successfully to characterize fuel moisture conditions [37]. This climatic metric has the potential to enable GIS-based spatial predictions of favorable future mosquito habitat conditions.

A major limitation associated with our study is related to the original case human occurrence data. These data on WNV human infections are case occurrences by zip code. This presented a spatial problem that could have been avoided by using address-specific occurrence data; however, due to patient confidentiality issues, these data were unavailable. For the same reason, social data such as income, age or race that could greatly improve the results of the modeling were not modeled in this study. Other potential sources of urban mosquito habitat such as ponds, flower pots, landfills, etc. were not considered in this study. Similarly, habitat suitability requirements for each mosquito species were not considered. Data necessary for these in-depth analyses of relatively small geographic areas and highly specific ecological conditions were not appropriate for this statewide study.

Additional modeling bias may exist due to various areal extents of zip codes. For example, rural zip codes in Mississippi tend to be larger, and can potentially include more heterogeneous ecological conditions. This could result in weakened mean comparisons for variable states in zip codes with WNV occurrence versus zip codes without WNV occurrence. The importance (weight) of stream density to the final landscape-base model may need to be reassessed in light of an apparent contradiction between certain mosquito species breeding sites (floodplains) and risk. Final risk models indicate relatively low risk in some of these floodplain areas. Typically, human settlement patterns on the first and second terrace positions near floodplains result in higher road density, lower stream density, and gentle slopes that favor *Culex *species. Our models do reflect high-risk conditions that result from the combined effects of those variable states in numerous areas adjacent to the floodplains.

Our results did not support the assertion that WNV is predominantly an urban problem, but indicated that WNV may be a problem for rural areas as well. Nevertheless, humans are mobile and no consideration was given to the possibility that infections occurred outside a zip code of residence. In a more specific study of individual cases, the travel habits of WNV infected individuals could be ascertained and the distance from home and travel frequency used as a stratification tool to filter model inputs to those human cases that occurred in close proximity to their homes or workplaces.

In summary, this research indicated that the assessment of WNV risk on a state level can be effectively performed using widely available environmental data combined with nonhuman surveillance information to support disease monitoring and prediction efforts. Our models were constructed in a desktop computing environment and can be easily implemented in an automated decision support system that may help public officials to be better prepared to combat this and other vector-borne diseases. Additive modeling gives a landscape-based detailed risk assessment at every cell location, which can be further summarized to show relative risk within areas that have distinct boundaries such as state parks, zip codes or recreation areas. This information can help to better define mosquito control strategies and help regulatory agencies to focus their prevention efforts. Finally, modeling disease with GIS results in spatial depictions of the risk that can be used as input to mosquito and bird sampling strategies designed for detection of WNV in the environment.

## Abbreviations

DEM – Digital Elevation Model

GIS – Geographic Information Systems

HRAP – Hydrologic Rainfall Analysis Project

IDW – Inverse Distance Weighting

LPDAA – Land Processes Distributed Active Archive Center

MARIS – Mississippi Automated Resource Information System

MODIS – Moderate Resolution Imaging Spectroradiometer

MPE – Multi-Sensor Precipitation Estimate

NDVI – Normalized Difference Vegetation Index

P-E – precipitation minus evaporation

SAW – simple additive weighting

VMR – variance to mean ratio

WNV – West Nile virus

## Competing interests

The author(s) declare that they have no competing interests.

## Authors' contributions

All authors made substantial contribution to the conceptual design, data acquisition and analysis as well as drafting the final version of the manuscript. WHC acquired project funding, was responsible for the conceptual design and administration of the project, participated in and supervised data analysis and interpretation, and has been involved in drafting the manuscript. KG carried out the analysis, developed final models, interpreted the results, designed figures and drafted the manuscript. RCW was responsible for data acquisition, variable preparation, preliminary analysis, and participated in manuscript writing. All authors read and approved the final version of submitted manuscript.
